# Cyborg psychiatry to ensure agency and autonomy in mental disorders. A proposal for neuromodulation therapeutics

**DOI:** 10.3389/fnhum.2013.00463

**Published:** 2013-09-05

**Authors:** Jean-Arthur Micoulaud-Franchi, Guillaume Fond, Guillaume Dumas

**Affiliations:** ^1^Unité de Neurophysiologie, Psychophysiologie et Neurophénoménologie, Solaris, Pôle de Psychiatrie Universitaire, Hôpital Sainte-MargueriteMarseille, France; ^2^Laboratoire de Neurosciences Cognitives, UMR CNRS 7291, 31 Aix-Marseille Université, Site St CharlesMarseille, France; ^3^Université Paris Est-Créteil, Pôle de Psychiatrie du Groupe des Hôpitaux Universitaires de Mondor, INSERM U955, Eq Psychiatrie Génétique, Fondation FondaMental Fondation de Coopération Scientifique en Santé MentaleParis, France; ^4^Equipe Cogimage (ex-LENA CNRS UPR 640), CRICM - UPMC/INSERM UMR-S975/CNRS UMR7225Paris, France; ^5^Human Brain and Behavior Laboratory, Center for Complex Systems and Brain Sciences, Florida Atlantic UniversityBoca Raton, FL, USA

**Keywords:** brain computer interface, neurofeedback, non-invasive brain stimulation, transcranial magnetic stimulation, brain-state-dependent stimulation, mental disorder, psychiatry, neurocognitive networks

## Abstract

Neuromodulation therapeutics—as repeated Transcranial Magnetic Stimulation (rTMS) and neurofeedback—are valuable tools for psychiatry. Nevertheless, they currently face some limitations: rTMS has confounding effects on neural activation patterns, and neurofeedback fails to change neural dynamics in some cases. Here we propose how coupling rTMS and neurofeedback can tackle both issues by adapting neural activations during rTMS and actively guiding individuals during neurofeedback. An algorithmic challenge then consists in designing the proper recording, processing, feedback, and control of unwanted effects. But this new neuromodulation technique also poses an ethical challenge: ensuring treatment occurs within a biopsychosocial model of medicine, while considering both the interaction between the patients and the psychiatrist, and the maintenance of individuals' autonomy. Our solution is the concept of Cyborg psychiatry, which embodies the technique and includes a self-engaged interaction between patients and the neuromodulation device.

## Non-invasive electrophysiological interventions in psychiatry

A new therapeutic approach in psychiatry is to modulate neural networks of the brain in order to induce neural plasticity (Peled, 2005; Linden, 2006; Schneider et al., 2009; Thut and Pascual-Leone, 2010). However, traditional treatments for mental disorders such as pharmacology and psychotherapy give little consideration to the neural network dynamics (Mackey and Milton, 1987). Psychiatric drugs could have long-term neuroplastic effects but are difficult to adapt to each patient (Fond et al., 2012). Psychotherapies, in particular Cognitive Behavior Therapy (CBT), have an adaptive and interactive effect on the brain but it remains quite indirect (Goldapple et al., 2004). Two non-invasive electrophysiological interventions, however, are proving promising in brain therapeutics for mental disorders:

- Electrical brain stimulations devices: Transcranial Magnetic Stimulation (TMS; Miniussi and Rossini, 2011) and Transcranial Direct Current Stimulation (tDCS; Polania et al., 2010)
- Neurofeedback (NF) devices (Coben and Evans, 2011).

Repeated TMS (rTMS) and NF are valuable therapeutics in the field of psychiatry (Yucha and Montgomery, 2008; Coben and Evans, 2011), but with rTMS we are confronted with the confounding effects of brain-mind states and, with NF, difficulties to change neural dynamics could be a potential problem. In the current proposal our aim is twofold: (i) to explain how rTMS and NF coupling may offer a solution to the two aforementioned problems, and (ii) to analyze how these neuromodulation techniques may be integrated into an individual's brain dynamics and conception of him or herself as an autonomous agent (Glannon, 2013). In effect, we argue that the coupling of rTMS and NF can pave the way for a direct, adaptive, and interactive brain therapy in which patients can be self-engaged.

### rTMS and the effects of brain-mind states

Repetitive transcranial magnetic stimulation (rTMS) comprises a non-invasive and painless way to induce magnetic flux activation (high frequency) or inhibition (low frequency; Lisanby et al., 2002). Efficient in the treatment of psychiatric disorders, it has proved particularly robust for the treatment of major depressive episode (MDE), and results of its use in schizophrenia are encouraging (Lisanby et al., 2002; Coben and Evans, 2011). rTMS modifies neuronal activity in the selected superficial brain structure, but also modulates neural network activity (Lisanby et al., 2002; Huerta and Volpe, 2009). Thus, basic research carried out on TMS has led to the concept of “state dependency TMS” (Silvanto and Pascual-Leone, 2008). This concept suggests that the activation states of the neural circuits both before and during the stimulation influence the pulse effect. Indeed, TMS effect must be seen, not simply as the result of an applied stimulus, but as the result of the interaction between the applied stimulus and the level of brain activity (Silvanto and Pascual-Leone, 2008). Thus, the effects of rTMS are dependent on the brain-mind states of the stimulated subject (Bonnard et al., 2003). Therefore, current high variability of therapeutic effects of rTMS in mental disorders may be due in part to its partial account of individuals' neurodynamics and its effects on distant neural sites, even with localized stimulations (Vedeniapin et al., 2010).

Basic research suggests that rTMS efficiency could be increased in psychiatric disorders by triggering patients' brain activities during stimulation (Micoulaud-Franchi et al., 2013). Thus “interactive rTMS protocols” have been proposed (Micoulaud-Franchi et al., 2013). In NeuroAnalysis 2008 (Peled) said: “a future potential ‘brain pacemaker’ would probably involve a multiple-coil TMS device coupled with an EEG-dependent feedback mechanism, similar to a cardiac pacemaker set to act according to the ECG arrhythmias” (Peled, 2008). Thus, a “brain pacemaker,” commonly referred to as “Brain-State-Dependent Stimulation” (BSDS; Walter et al., 2012), would comprise an adaptive TMS coupled to the ongoing brain activity; the stimulation would vary in time, intensity, frequency, and topography according to an on-line EEG feedback. EEG coupled TMS is “a technique that has come of age” (Fitzgerald, 2010) and offers new possibilities for the treatments of mental disorders (Thut and Pascual-Leone, 2010; Miniussi and Vallar, 2011). Price et al. show the first encouraging results of the use of this kind of adaptive/contingent rTMS in the treatment of MDE (Price et al., 2010).

### Neurofeedback and the difficulties to change neural dynamics

NF is a non-invasive technique that enables an individual to learn the cognitive strategies required to change neurophysiological activity (i.e., EEG), for the purposes of improving health and performance (Yucha and Montgomery, 2008). The originality of NF is that it gives patients a more active role in there own health care (Yucha and Montgomery, 2008) and comprises a holistic conception in which cognitive and brain activities are modified together (Rémond, 1997; Cherici and Barbara, 2007; Coben and Evans, 2011). For this reason, NF is also referred to as “psychoneurotherapy” (Paquette et al., 2009), “brain psychotherapy” (Micoulaud-Franchi and Vion-Dury, 2011) or “neuroimagery therapy” (deCharms, 2008). Indeed, NF facilitates an on-line self-regulation of brain activity and as such may be considered as an adaptive and interactive brain therapy (Micoulaud-Franchi et al., 2012).

However, for certain subjects, modifying their neural dynamics through NF can prove very difficult. In a NF study aimed at investigating to what extent the regulation of excitability in cortical networks is impaired in epileptic patients, it was found that performance on NF was initially below healthy subjects and that “not every patient seemed to be able to achieve this control” (Rockstroh et al., 1993). This difficulty is also found in the field of Brain Computer Interface (BCI). BCI was developed, in particular, as assistive technology for patients with motor disabilities (Wang et al., 2010). BCI is commanded directly by brain activity feedback (EEG, MEG or fMRI activities measurements), with EEG activity constituting the most commonly used brain activity feedback. However, BCI performances show large variability across individuals, and for a non-negligible proportion of users (estimated at 15–30%), BCI control does not work (Vidaurre and Blankertz, 2010).

Many solutions have been proposed to optimize NF and BCI. Solutions based “on the participants” are closed to cognitive and behavioral therapeutics. The aims are to enhance the motivation of the participants, to help the participants to try different strategies, to explicit individual-specific control strategies and to apply the learned self-regulation skills in real-life situations (Kotchoubey et al., 2001). Solutions based “on the BCI loop,” were proposed to optimize BCI performance. We suggest that some of these solutions could be applied to optimize NF for treatment of mental illness. The first involves an algorithmic solution that aims to develop a machine-learning mechanism (Vidaurre and Blankertz, 2010). It is in line with the concept of co-adaptation in which the tool becomes functionally involved in the extraction and definition of the user's goals: both subject and the tool are learning (Sanchez et al., 2009). The second solution comprises a “hybrid BCI,” in which two BCIs are combined, for example: event-related (de)synchronization (ERD, ERS) of sensorimotor rhythms and steady-state visual evoked potentials (SSVEP; Pfurtscheller et al., 2010). The third solution comes from basic research in animals and invasive BCI. It uses closed-loop neural interface technology that combines neural ensemble decoding with simultaneous electrical microstimulation feedback (Marzullo et al., 2010; Mussa-Ivaldi et al., 2010). However, very few studies have used this solution to optimize BCI in humans (Walter et al., 2012). Birbaumer suggested: “The combination of these stimulation techniques (TMS, tDCS, neurochips) with BCIs is a largely unexplored field” (Birbaumer and Cohen, 2007), and, at the same time, research has yielded encouraging results showing that TMS may help participants to increase their brain EEG response performance in BCI (Kubler et al., 2002; Karim et al., 2004). This solution is, therefore, worthy of interest in the field of NF in psychiatry. Indeed, recurrent neuronal networks have been used to propose an interpretation of several mental dysfunctions (Pezard and Nandrino, 2001), which is evidence in itself that it is particularly difficult to modify one's brain activity when one has such mental disorders. Thus, rTMS could bring the necessary energy to break the recurrent neural network dynamics in order to help the patient explore new neural network dynamics and, by means of the NF device, change his/her EEG activity in the desired way to improve health and performance (Micoulaud-Franchi and Vion-Dury, 2011). tDCS may also enhance the effect of cognitive remediation techniques (Andrews et al., 2011) and could, thus, have the same positive effect on NF (Miniussi and Vallar, 2011).

## Coupling non-invasive electrophysiological interventions

### The challenge of closing the loop

**Figure 1:**
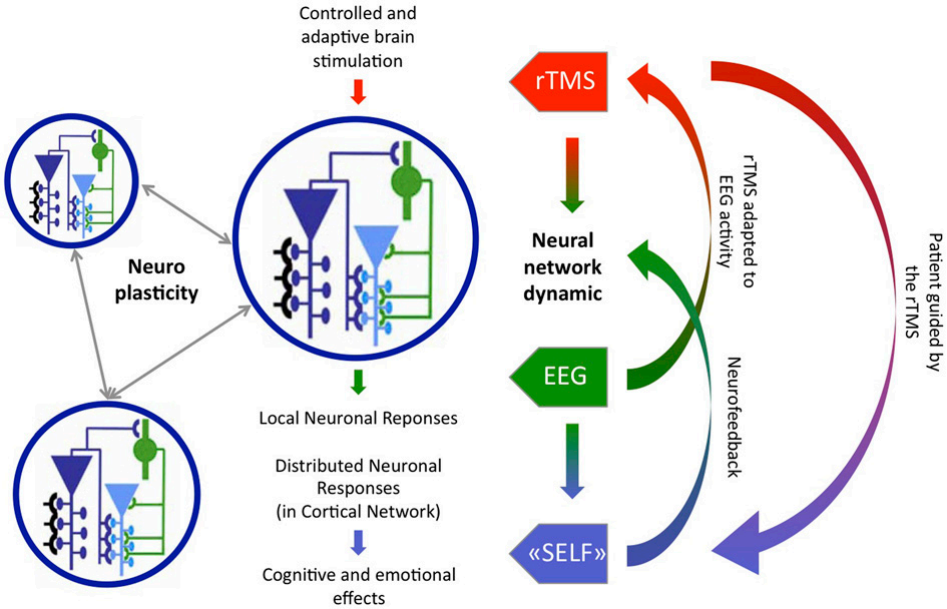
**Combination of Brain-State-Dependent Stimulation (green-red loop) and Neurofeedback rTMS (blue-red loop) as an example of Cyborg psychiatry device, adapted from Thut and Pascual-Leone (2010)**.

To summarize, firstly TMS may be improved by taking into account brain activity (particularly EEG activity) to stimulate the brain (Price et al., 2010) and, secondly, NF could be improved by combining it with TMS or tDCS brain stimulation (Kubler et al., 2002). In addition, further research needs to be undertaken in this area to replicate the preliminary results in mental disorders (Price et al., 2010). However, here we propose to investigate the challenge of neuromodulation techniques that couple these two aforementioned improvements. We previously proposed the concept of “Neurofeedback rTMS” (Micoulaud-Franchi and Vion-Dury, 2011): in which the rTMS efficacy is enhanced by the background EEG, which is self-regulated by subjects through NF, and, at the same time, the subject is helped by the rTMS to create this background EEG. TMS/tDCS-NF coupling can, therefore, close the loop completely in order to optimize simultaneously the non-invasive neurostimulation techniques and the NF, See Figure 1. TMS/tDCS-NF coupling is, however, confronted by two challenges: the first is algorithmic, the second is ethic.

The algorithmic challenge involves determining the kind of brain activity that will be recorded and the kind of feedback that will be made, how all these data will be treated in real time and how to control unwanted effects. The first issue is related to the use of a new diagnostic system correlated to the neural network disturbance in mental disorders. The “Clinical Brain Profiling” advanced by Peled is an interesting approach to these novel therapeutic hypotheses based on TMS/tDCS-NF coupling (Peled, 2006, 2009). Peled proposed a new etiology-oriented diagnostic system for psychiatry based on neural network dynamics complexity and neural plasticity (Peled, 2004). It provides an innovative heuristic for recording brain activity and will soon integrate data from TMS-EEG research (Ilmoniemi et al., 1997; Thut and Pascual-Leone, 2010). The second issue related to such approaches is how to better account for non-linear dynamics in neuroscience. This is already being tackled at the theoretical levels, but relies, also, on the development of new methods. One such novel method is the “dynamic clamp” advanced by Prinz et al. (2004), which consists in dynamically interfacing living cells with their simulated counterpart. This technique creates a “hybrid network” incorporating the inherent nonlinearities of most physiological processes (Prinz et al., 2004). Such a concept has been already scaled from the neural to the behavioral scale with the so-called “Virtual Partner Interaction” (VPI; Kelso et al., 2009). VPI could constitute a paradigmatic model for the therapeutic approach described in the current paper (Werry et al., 2001). The last algorithmic issue is related to some problems appearing in closed loop systems (Corke and Good, 1996). Indeed, a closed loop feedback system based on NF and rTMS/tDCS could lead to unforeseeable “resonance” effects in the brain that should be investigated and be taken into account.

The ethic challenge is in line with the general aim of psychiatry, which tries to enable patients to lead a more self-determined life. Indeed, psychiatry increasingly uses neuromodulation techniques in the treatment of mental disorders. For example, the Mind Machine Project (MMP) initiated in 2009 by the Massachusetts Institute of Technology (MIT) is “looking for advanced applications of these technologies, such as “non-chemical based” solutions for psychiatric treatments and brain prostheses.” In addition the concept of neurorehabilitation has been applied in the field of psychiatry (Bach-Y-Rita, 2003; Miniussi and Rossini, 2011; Miniussi and Vallar, 2011). Thus, the question is: how can we ensure that all these techniques restore or enhance a person's agency and autonomy? Related to this, we propose a first ethical issue based on the biopsychosocial model of medicine and a third person perspective (Glannon, 2013). This issue is related to the fact that these neuromodulation techniques depend on the interaction between the learner (subjects) and the trainer (practitioner/therapist), and are constructed as a process that occurs within a biopsychosocial context and social constraints (Glannon, 2013). We also put forward a second, more radical, ethical issue based on a neurophenomenological point of view and a first person perspective. Here we suggest that agency and autonomy depend on the capacity of all these techniques to be embodied by the patients. Such an approach is already present in closed loop technology for sensory substitution (Bach-Y-Rita, 2003; Bach-Y-Rita and Kercel, 2003). The ethical issue is ensured by the fact that the subject used the device as a part of his/her body. The device has to open up a world to the subject that will be appropriated by himself or herself. Similarly, TMS/tDCS-NF coupling could help patients to promote therapeutic neural plasticity using their own brain connectivity and without the direct intervention a third party (Linden, 2006; Schneider et al., 2009). Of course, psychiatrists should still help the patients, but the important point is that the device enables the subject to rediscover their own mind-brain world and from their own first person perspective. This ethical point of view leads us to the concept of Cyborg.

### Back to the cyborg concept as an heuristic for cutting across mind, brain and devices

“Cyborg” is a term coined in 1960s, in the context of the challenges presented by space flight and travel, with the famous article entitled “Cyborgs and Space,” by Kline, a psychiatrist at Rockland State Hospital, and Clynes, a scientist at the Dynamic Simulation Lab (Clynes and Kline, 1960; Gray, 1995). “Cyborg” combined the words “cybernetic” and “organism.” The concept involves devices that enable an organism to live outside its habitat (in this case: Space): “The Cyborg deliberately incorporates exogenous components extending the self-regulatory control function of the organism in order to adapt it to new environments” (Clynes and Kline, 1960). Consequently, a Cyborg is a kind of extended embodiment, an organism that is, at the same time, natural and artificial, and, as such, questions the limits between organism, technology and external environment (Tomas, 1995).

In 1970, Clynes wrote, this time without Kline, a second Cyborg article entitled “Sentic space travel” (Clynes, 1995). This Sentic Cyborg involves devices that enable a human “to express his emotion in accordance with his nature” to enable them to carry out very long space-flights (Gray, 1995). Initially refused, Clynes' proposition is now of theoretical interest in light of the new possibilities of cognitivo-brain modulation using TMS/tDCS and NF. Kline and Clynes' original question, “What are some of the devices necessary for creating self-regulating man-machine systems (…) to unconsciously adapt it to new environments?” (Clynes and Kline, 1960), can now be rephrased as: What are the devices needed to create self-regulating brain-machine systems to be used by patients with mental disorders to promote new brain/mind dynamics? By extending the first Cyborg hypothesis of Kline and Clynes, the new direct, adaptive, and interactive brain therapies proposed in this paper could not only open the door to new ways of interacting with the outside (Space), but also create new possibilities of dealing with the inside (brain-mind).

As Clynes suggested in the conclusion of his Sentic Cyborg hypothesis: “Through understanding our unconscious heritage consciously, we may be able to teach our automatic systems to live in harmony with our old heritage, as well as with our new exploration of outer, and perforce, inner, space” (Clynes, 1995). The benefit of the cyborg hypothesis is that it leads the psychiatrist to consider neurostimulation techniques (as TMS or tDCS), not just as an outside brain constraint, but also as a brain guidance interaction in which the patient's mind is self-engaged. This hypothetical point of view is meanly theoretical and need to be tested with some experimental observations in order to confirm its effectiveness and its lack of unwanted and side effects as “resonant” effects (Corke and Good, 1996). However, we wanted to stress that the future of neuromodulation treatments for mental disorders will involve dealing, firstly, with neural network dynamics (Peled, 2006, 2008) and, secondly, with the capacity of the treatment to exploit the point of view of the patients, to act as a cyborg device.

### Conflict of interest statement

The authors declare that the research was conducted in the absence of any commercial or financial relationships that could be construed as a potential conflict of interest.
